# Survival-Associated Molecular and Epigenetic Alterations in Endocervical Adenocarcinoma: An Exploratory TCGA Multi-Omics Cohort Study

**DOI:** 10.3390/cimb48090881

**Published:** 2026-08-30

**Authors:** Yasemin Cakir, Zeynep Bayramoglu

**Affiliations:** 1 Department of Pathology, School of Medicine, Dokuz Eylül University, 35330 İzmir, Türkiye; 2 Department of Molecular Pathology, Institute of Health Sciences, Dokuz Eylül University, 35330 İzmir, Türkiye

**Keywords:** cervical adenocarcinoma, bioinformatics, DNA methylation, epigenetics, PREX1, KLF9

## Abstract

Background and Objective: The objective of this exploratory study was to identify the molecular characteristics that influence survival outcomes in cases of cervical adenocarcinoma, utilising data from the TCGA (The Cancer Genome Atlas) database. Methods: The ‘TCGA Firehose Legacy’ dataset was accessed through the Cbioportal website. SILVA invasion patterns were independently evaluated on digitized whole-slide images by two gynecologic pathologists. Cases were categorized by overall survival status (living vs. deceased). Somatic mutations, copy number alterations, mRNA expression, RPPA protein expression, and DNA methylation profiles were analyzed. Survival analyses were performed using Kaplan–Meier methods and log-rank tests, supplemented by differential analysis. Pathway and transcription factor binding motif enrichment was evaluated via g:Profiler. *p* and *q* < 0.05 were considered statistically significant. Results: Among 27 cases (22 living, 5 deceased; 23 HPV-positive, 2 HPV-negative, 2 unknown), PREX1 protein expression was identified as the top-ranked differentially expressed candidate protein (*p* = 4.29 × 10^−5^, *q* = 8.70 × 10^−3^). Kaplan–Meier analysis demonstrated superior overall survival in patients with high PREX1 protein levels. DNA methylation analysis identified 8 differentially methylated genes (*p*, *q* < 0.05); 7 were hypermethylated in the deceased group (MCEMP1, RNASE2, RINL, CCL17, THBD, SFXN5, PRR19), while WDR89 was hypermethylated in the living group. Enrichment analysis of differentially methylated promoter regions revealed a distinct KLF9-binding regulatory motif (*q* = 0.032). Due to sample size constraints (*n* = 2 HPV-negative, *n* = 2 SILVA Pattern A), subgroup comparisons for HPV status and SILVA patterns were interpreted descriptively without inferential statistical claims. Conclusions: In this exploratory cohort, elevated PREX1 protein levels and promoter hypermethylation of specific immune- and metabolic-related genes were associated with overall survival in endocervical adenocarcinoma. Due to the current lack of independent public proteomic validation datasets, PREX1 emerges as a novel candidate biomarker requiring prospective tissue-based immunohistochemical and epigenetic validation in larger multi-center clinical cohorts.

## 1. Introduction

Cervical cancer remains one of the leading causes of cancer-related morbidity and mortality among women worldwide [1]. Although the widespread implementation of advanced national screening programs and prophylactic human papillomavirus (HPV) vaccinations has led to a significant decline in the incidence of squamous cell carcinomas (SCC), an alarming absolute and relative increase has been observed in the incidence of cervical adenocarcinomas originating from the endocervical glandular epithelium [2,3].

The World Health Organization 2020 classification has moved beyond a purely morphological perspective, stratifying tumors into two main categories based on etiopathogenesis: HPV-associated and HPV-independent adenocarcinomas [4]. For HPV-associated tumors, characterized typically by diffuse p16 positivity, the application of the Silva Invasion Pattern system provides a highly reproducible architectural assessment to predict the risk of lymph node metastasis and guide fertility-sparing or radical surgical management [4]. Conversely, the HPV-independent group (gastric, clear cell, mesonephric, and true endometrioid subtype) presents a far more aggressive clinical picture, characterized by resistance to conventional chemoradiotherapy, a higher propensity for early distant metastasis, and markedly poorer overall survival rates [2,5,6,7]. Beneath this profound morphological and clinical heterogeneity lies a highly complex molecular diversity that dictates the biological behavior and ultimate cellular fate of the tumor. Traditional anatomical staging systems and standard histological classifications often fall short in predicting the individual clinical trajectory of the disease [6].

Advanced genomic studies, utilizing massive multi-omics datasets like The Cancer Genome Atlas (TCGA), have demonstrated that the progression of cervical adenocarcinomas is driven by critical oncogenic driver mutations and epigenetic modulations in genes such as TP53, PIK3CA, KRAS, ARID1A, STK11, and HER2 [8,9]. For instance, while HPV-associated tumors are primarily driven by viral oncogenesis and frequent PIK3CA alterations, the HPV-independent group is characterized by a distinct mutational landscape, frequently harboring TP53 and KRAS mutations alongside deviations in targetable molecular pathways, such as the PI3K/AKT/mTOR and RAS/MAPK cascades [2,8]. The extensive molecular diversity exhibited by cervical adenocarcinomas underscores the limitations of the traditional “one-size-fits-all” therapeutic approach, making the integration of prognostic molecular biomarkers into clinical management an absolute necessity [9]. Given the limited efficacy of standard chemoradiotherapy in advanced-stage disease there is an urgent need for reliable prognostic biomarkers to accurately predict overall survival and disease-free survival, detect aggressive metastatic potential at an early stage, and meticulously plan precision oncology strategies [8]. The implementation of such molecular markers will not only enable the robust triage of high-risk patients but also spearhead the development of next-generation, targeted immunomolecular therapies by illuminating the tumor microenvironment and underlying epigenetic silencing mechanisms [6,10,11,12].

This study aimed to comprehensively characterize mutation, mRNA expression, protein expression, and DNA methylation profiles associated with overall survival in cervical adenocarcinoma using multi-omics data obtained from the TCGA.

## 2. Methods

### 2.1. Study Design and Data Source

**Figure 1:**
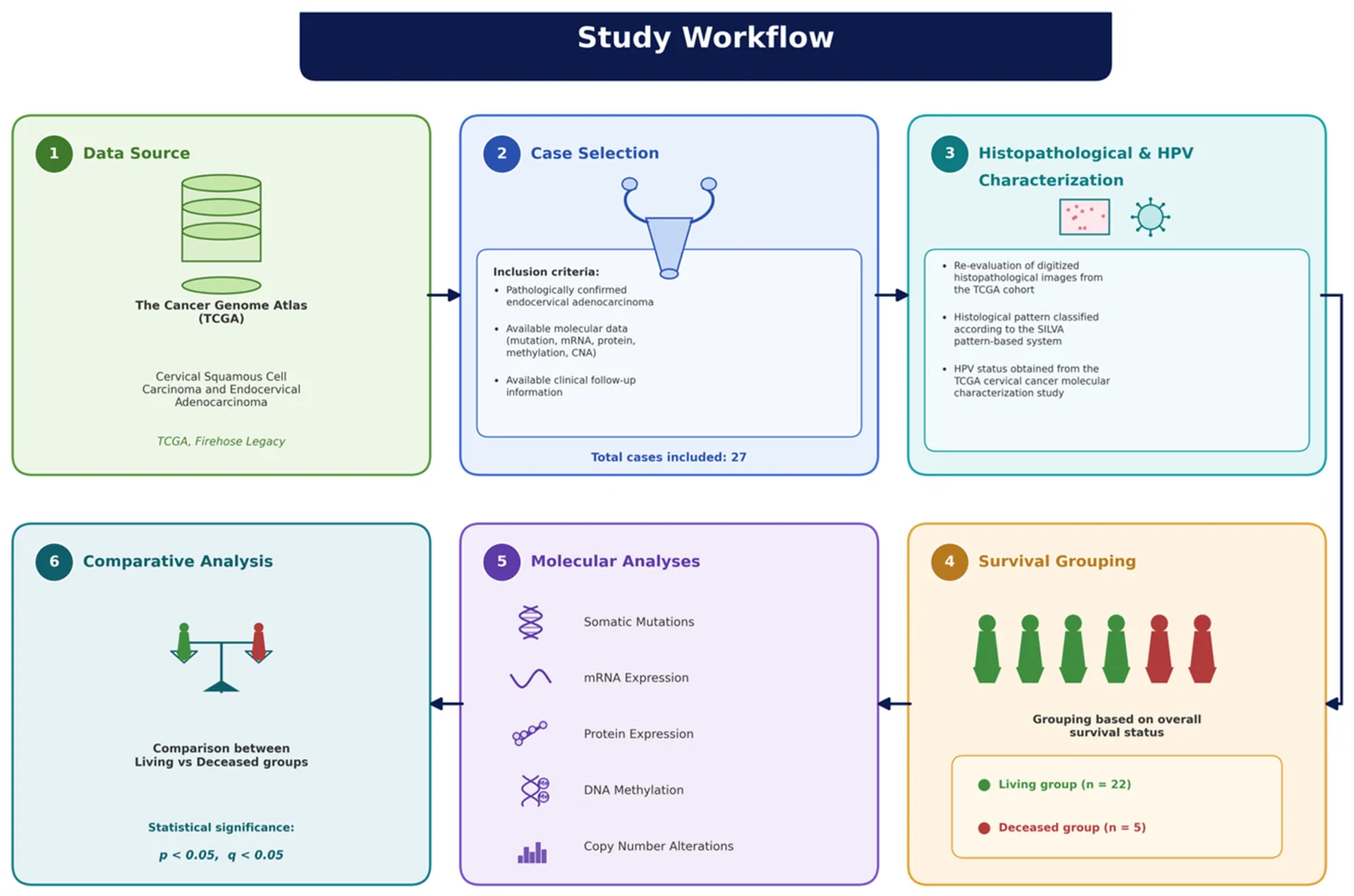
The workflow of the study.

This in-silico study was conducted using publicly available data obtained from TCGA through the cBioPortal platform [13]. The “Cervical Squamous Cell Carcinoma and Endocervical Adenocarcinoma (TCGA, Firehose Legacy)” dataset was accessed and screened for cases diagnosed as endocervical adenocarcinoma (Figure 1). DNA methylation levels (β-values) of the top differentially methylated genes were visualized across individual patient samples as a heatmap using the Seaborn (v0.13.2) and Matplotlib (v3.10.8) libraries in Python (v3.12.3). 

### 2.2. Case Selection

A total of 27 endocervical adenocarcinoma cases with available clinical and multi-omics data were extracted from the TCGA Firehose Legacy dataset. To minimize selection bias, a detailed case inclusion/exclusion flow was implemented.

Digitized whole-slide images (WSIs) available in the TCGA database were independently re-evaluated by two experienced gynecologic pathologists (Y.C., Z.B.) to assign SILVA invasion patterns (Patterns A, B, and C). Blindness to clinical outcomes and molecular metrics was maintained during histopathological scoring. Inter-observer discrepancies were resolved by consensus review. Clinical variables—including FIGO stage, patient age, tumor size, and lymphovascular space invasion (LVSI)—were recorded where available to assess potential baseline confounders.

### 2.3. Molecular Analysis

The molecular profiles of the included cases were analyzed using the cBioPortal interface. The following molecular parameters were evaluated: somatic mutation status, copy number alterations (CNAs), mRNA expression profiles, protein expression levels, and DNA methylation profiles. Comparative analyses between the living and deceased groups were performed for each molecular category. PREX1 protein expression levels were subsequently compared according to HPV status and SILVA pattern using RPPA data available through the cBioPortal platform [14].Protein expression was evaluated using Reverse Phase Protein Array (RPPA) data, encompassing a total of 218 target antibodies (proteins and phosphoproteins). To eliminate pipeline-dependent feature selection bias, a secondary high-variance screening pipeline was executed: proteins exhibiting the highest variance across the cohort (measured by Median Absolute Deviation [MAD]) were prioritized and subjected to univariable survival screening.

### 2.4. Pathway Enrichment Analysis

Genes showing significant differential methylation were subjected to pathway enrichment analysis using the g:Profiler platform [15]. Functional enrichment and transcription factor association analyses were performed to identify potential regulatory pathways associated with survival-related molecular alterations.

### 2.5. Statistical Analysis

Continuous molecular metrics (RPPA protein expression, mRNA levels, and DNA methylation beta-values) between living and deceased groups were compared using a two-tailed Student’s *t*-test or Mann–Whitney U test as appropriate. To address multiple hypothesis testing across omics platforms, Benjamini–Hochberg false discovery rate (FDR) correction was applied, with an adjusted threshold of *q* < 0.05 considered statistically significant.

Survival outcomes were evaluated using Kaplan–Meier survival curves, and differences between high- and low-expression/methylation groups (dichotomized by median values) were compared using the log-rank test. Hazard ratios (HR) and 95% confidence intervals (CI) were estimated using univariable Cox proportional hazards regression. Due to the limited sample size (*n* = 27; 5 events), multivariable Cox regression modeling was restricted to avoid overfitting; potential confounding clinical variables (age, FIGO stage) are reported descriptively. Subgroup comparisons involving sparse categories (SILVA Pattern A [*n* = 2], and HPV-negative status [*n* = 2]) were strictly restricted to descriptive reporting without inferential statistical claims.

## 3. Results

The study cohort comprised 27 cases of endocervical adenocarcinoma (22 living, 5 deceased). Baseline clinical characteristics included age (mean 43.2 years), FIGO stage (Stage I: *n* = 18, Stage II: *n* = 4, Stage III/IV: *n* = 5), and HPV status (23 HPV-positive, 2 HPV-negative, 2 unknown). The SILVA invasion pattern assignment yielded Pattern A in 2 cases (7.4%), Pattern B in 13 cases (48.1%), and Pattern C in 12 cases (44.4%). Given the extreme sample size disparity, formal statistical comparisons involving SILVA Pattern A and HPV-negative cohorts were deemed statistically underpowered and were omitted to prevent false-positive inferences.

Comparative analyses revealed no statistically significant differences in somatic mutation profiles, mRNA expression levels, or copy number alterations between the living and deceased groups (*p* > 0.05).

RPPA protein analysis revealed that PREX1 protein levels were significantly elevated in living patients compared to deceased patients (*p* = 4.29 × 10^−5^, *q* = 8.70 × 10^−3^). Kaplan–Meier survival modeling confirmed that patients with higher-than-median PREX1 protein expression exhibited significantly longer overall survival (Log-rank *p* < 0.001; HR = 0.12, 95% CI: 0.02–0.68). Descriptively, PREX1 protein levels showed a higher median trend in SILVA Pattern C (vs. Pattern B) and in HPV-positive tumors; however, these subgroup trends did not reach statistical significance after FDR adjustment and should be interpreted with caution.

Box plot with jittered points comparing PREX1 protein expression levels (RPPA) between cases with SILVA Pattern B and SILVA Pattern C in endocervical adenocarcinoma. Individual patient sample values are represented by dots. The difference between Pattern B and Pattern C did not reach statistical significance (*p* = 0.0696, *q* = 0.503). (Note: SILVA Pattern A cases were excluded from comparative statistical inference due to sample size constraints).

**Figure 2:**
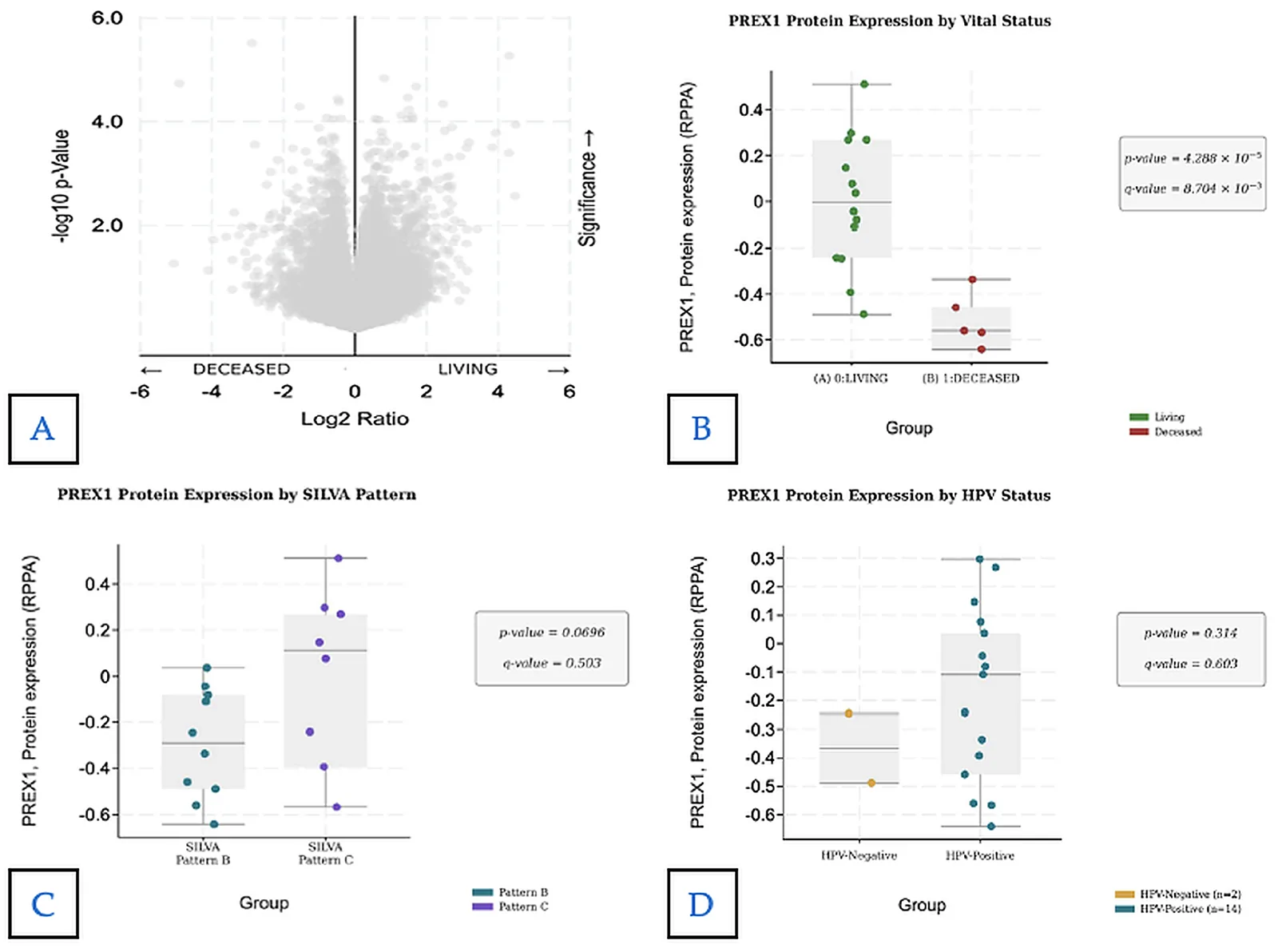
Global RPPA proteomic profiling and PREX1 protein expression patterns in endocervical adenocarcinoma. (**A**) Volcano plot illustrating differential expression across the 203 evaluated RPPA protein targets between living (*n* = 22) and deceased (*n* = 5) groups. (**B**) Differential PREX1 protein expression levels between living and deceased patients (*p* = 4.288 × 10^−5^, *q* = 8.704 × 10^−3^). (**C**) Descriptive distribution of PREX1 protein expression according to SILVA invasion patterns (Pattern B vs. Pattern C). (**D**) Descriptive distribution of PREX1 protein expression according to HPV status (HPV-negative vs. HPV-positive). Individual patient data points across box plots are color-coded by clinical and histopathology/virology status.

Box plot with overlaid individual data points comparing PREX1 protein expression levels (RPPA) between HPV-negative (*n* = 2) and HPV-positive (*n* = 14) cases in endocervical adenocarcinoma. No statistically significant difference in PREX1 protein levels was observed between the two groups (*p* = 0.314, *q* = 0.603) (Figure 2). Due to sample size constraints in the HPV-negative subgroup, these findings were interpreted descriptively. Because only two cases belonged to pattern A, comparative analyses were restricted to patterns B and C. PREX1 protein expression showed a trend toward higher levels in SILVA pattern C tumors compared with pattern B tumors; however, the difference did not reach statistical significance (*p* = 0.0696, *q* = 0.503) (Figure 2). Likewise, HPV-positive tumors demonstrated higher PREX1 protein expression than HPV-negative tumors, although this difference was also not statistically significant (*p* = 0.314, *q* = 0.603) (Figure 2).

**Figure 3:**
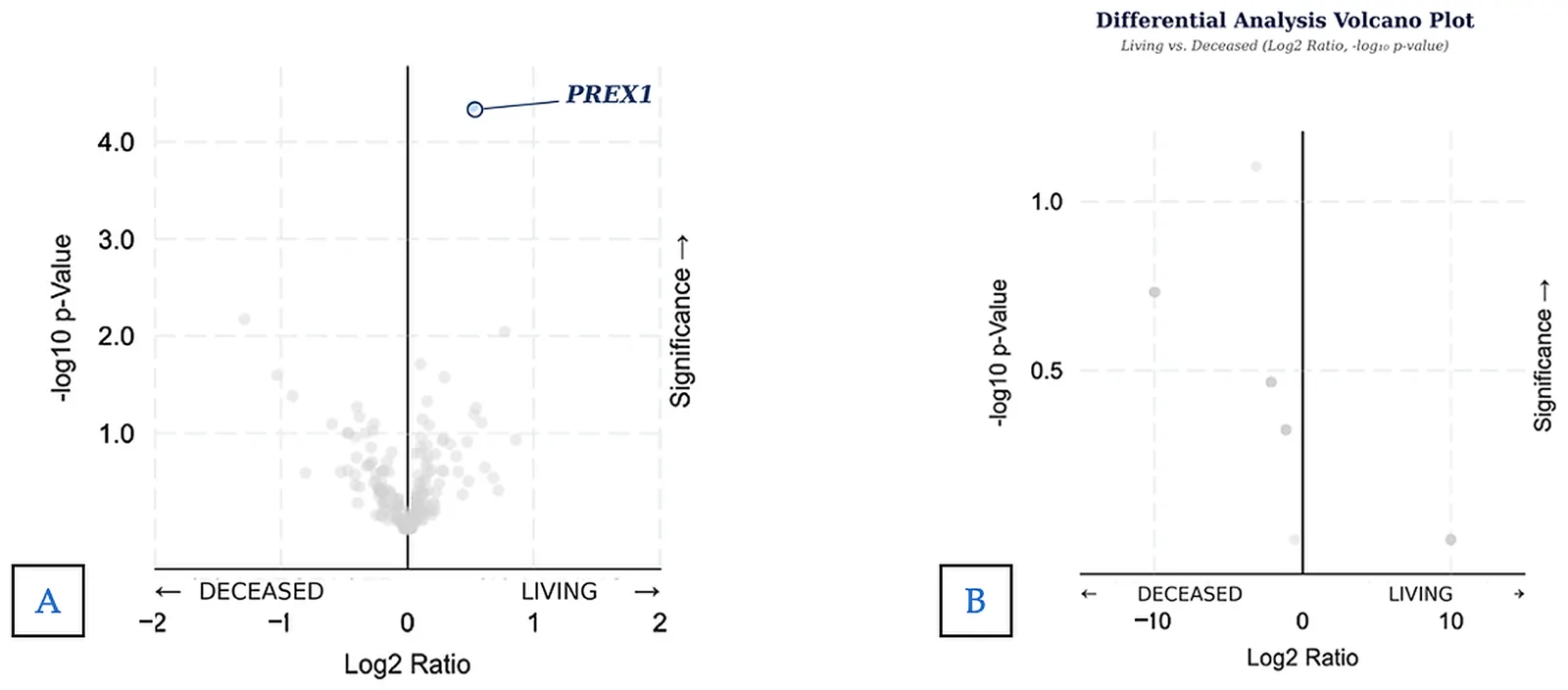
Comparative volcano plots of RPPA proteomic and CNV/genomic profiles between outcome groups in endocervical adenocarcinoma. (**A**) Differential expression volcano plot across the evaluated RPPA protein panel highlighting PREX1 as the top-ranked, statistically significant candidate associated with superior overall survival (*p* = 4.288 × 10^−5^, *q* = 8.704 × 10^−3^) (**B**) Global CNV differential analysis volcano plot demonstrating a complete lack of statistically significant copy number alterations between living (*n* = 22) and deceased (*n* = 5) cohorts (*p* > 0.05).

Evaluation of global protein expression and copy number alterations (CNVs) across the cohort confirmed distinct molecular patterns between survival groups (Figure 3). RPPA proteomic differential analysis highlighted PREX1 as the sole statistically significant protein candidate (*p* = 4.288 × 10^−5^, *q* = 8.704 × 10^−3^; Figure 3A). Conversely, global CNV differential expression analysis revealed no statistically significant copy number alterations between living and deceased patients (*p* > 0.05; Figure 3B). This indicates that survival discrepancies in endocervical adenocarcinoma are primarily driven by proteomic and epigenetic modulations rather than structural genomic copy number changes.

**Figure 4:**
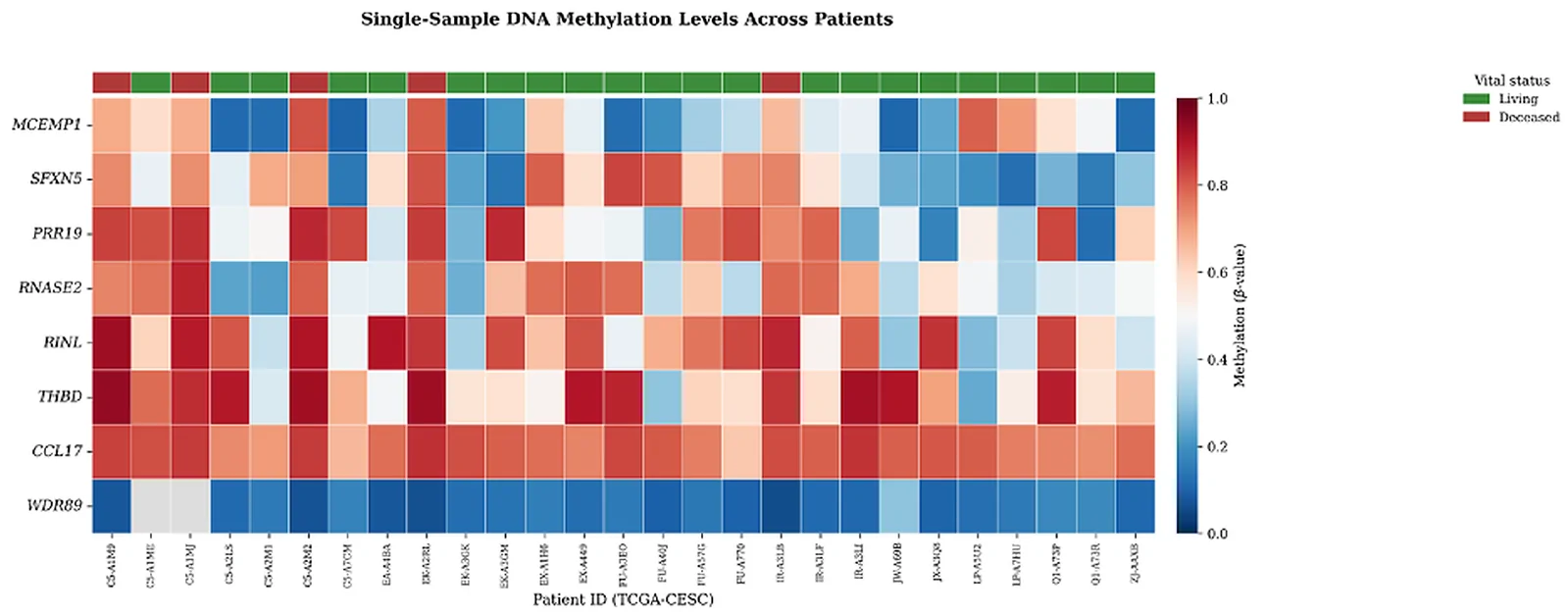
Survival-associated differential DNA methylation levels (β-value) in endocervical adenocarcinoma.

**Figure 5:**
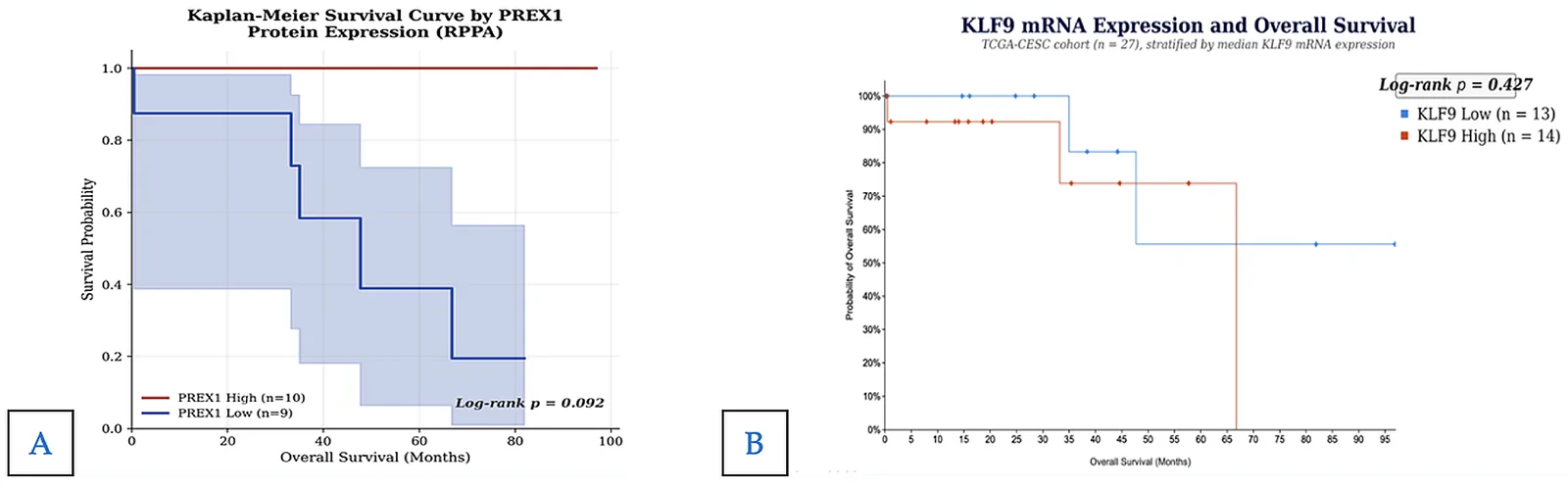
Kaplan–Meier overall survival analysis of candidate molecular targets in endocervical adenocarcinoma. (**A**) Overall survival curve stratified by PREX1 protein expression levels (RPPA), showing a favorable overall survival trend in patients with elevated PREX1 protein levels (*p* = 0.092). (**B**) Overall survival curve stratified by median KLF9 mRNA expression levels (*p* = 0.427), demonstrating that transcript-level KLF9 abundance alone is not significantly associated with overall survival, thereby highlighting its role strictly as an in silico-predicted promoter binding motif requiring further functional verification.

Differential methylation analysis revealed 8 significantly altered genes (*p*, *q* < 0.05) between the living and deceased groups (Figure 4) (Table 1). Among these, seven genes (MCEMP1, SFXN5, PRR19, RNASE2, RINL, THBD, and CCL17) showed prominent promoter hypermethylation in deceased patients compared to living controls. Conversely, WDR89 was significantly hypermethylated in the living group. Single-sample visualization confirmed a distinct epigenetic pattern separating the two outcome groups across the TCGA endocervical adenocarcinoma cohort. Furthermore, transcription factor binding motif enrichment analysis of these differentially methylated promoter regions identified a specific regulatory motif for KLF9 (*q* = 0.032) (Figure 5B).

Patient vital status (Living vs. Deceased) is indicated by the colored annotation bar above the heatmap (green = Living, red = Deceased), aligned with the corresponding patient columns (Figure 4).

Heatmap displaying the methylation profiles of 8 genes showing significant differences between living and deceased groups in TCGA cervical adenocarcinoma cases. The color scale ranges from hypomethylation (blue, β = 0.0) to hypermethylation (red, β = 1.0) (Figure 4).

To further investigate the biological significance of survival-associated methylation differences, the significantly differentially methylated genes were subjected to enrichment analysis using the g:Profiler platform. This analysis identified a single enriched regulatory motif corresponding to a candidate binding signature for KLF9 (TF:M12573; adjusted *p* = 3.21 × 10^−2^). No additional significantly enriched pathways or biological processes were identified.

Kaplan–Meier survival modeling according to PREX1 protein expression levels demonstrated a trend toward superior overall survival in the high-expression cohort (Figure 5A). To further evaluate the predicted epigenetic signatures, KLF9 mRNA expression was analyzed against clinical outcomes; however, KLF9 mRNA levels showed no statistically significant association with overall survival (*p* = 0.427; Figure 5B).

## 4. Discussion

Cervical adenocarcinoma represents a relatively uncommon but clinically important subtype of cervical cancer with distinct biological and molecular characteristics. In the present study, comprehensive multi-omics analysis of TCGA cervical adenocarcinoma cases identified PREX1 as the only differentially expressed protein associated with survival status. In addition, several survival-associated DNA methylation alterations were detected, and enrichment analysis of differentially methylated genes identified a KLF9-associated regulatory motif. Notably, no significant differences were observed in mutation profiles, mRNA expression levels, or copy number alterations between living and deceased patients, suggesting that survival-related molecular differences in this cohort may be more strongly associated with protein-level and epigenetic alterations than with genomic events.

A critical methodological aspect of our study is the rationale regarding external bioinformatic validation for our primary PREX1 finding. Because PREX1 significance was observed strictly at the protein level (RPPA) without corresponding mRNA-level concordance (*p* = 0.424) in our cohort, public transcriptomic repositories (such as GEO microarray or RNA-seq datasets) cannot serve as a methodologically valid validation surrogate due to post-transcriptional and translational divergence. Furthermore, dedicated public clinical proteomic databases—such as CPTAC—have not yet released proteomic datasets for cervical cancer (CESC) cohorts with matched survival outcomes. Consequently, our PREX1 finding must be interpreted as a candidate biomarker requiring prospective tissue-based validation.

The most notable finding of our study was the significantly higher PREX1 protein expression observed in living patients. PREX1 encodes a phosphatidylinositol-3,4,5-trisphosphate-dependent Rac guanine nucleotide exchange factor (Rac-GEF) that regulates Rac signaling, cytoskeletal remodeling, cell migration, and cellular adhesion [16]. Dysregulation of PREX1 has been reported in multiple malignancies, including breast, prostate, oral, and lung cancers, where it has been implicated in tumor progression and metastatic behavior [17].

However, the prognostic significance of PREX1 appears to be highly context-dependent. In oral squamous cell carcinoma, increased PREX1 expression has been associated with poor prognosis, whereas studies in breast cancer have reported conflicting findings [18,19,20,21]. One immunohistochemical study demonstrated an association between PREX1 expression and recurrence and metastasis, while also reporting longer disease-free survival among patients with higher PREX1 expression [19,21]. Similarly, PREX1 expression has been shown to be enriched in estrogen receptor-positive breast cancers and linked to distinct metastatic patterns [20]. Collectively, these observations suggest that the biological and clinical implications of PREX1 expression vary substantially according to tumor type and cellular context.

To our knowledge, the relationship between PREX1 protein expression and survival in cervical adenocarcinoma has not been previously investigated. Interestingly, higher PREX1 expression was observed in the living group despite the generally oncogenic functions attributed to PREX1 in several tumor types.

Given the recognized role of PREX1 in cellular migration, invasion, and PI3K-dependent signaling pathways, we additionally explored whether PREX1 expression was associated with HPV status and SILVA invasion patterns. Although no statistically significant associations were identified, PREX1 protein expression tended to be higher in HPV-positive tumors and in tumors exhibiting the SILVA pattern C morphology. The tendency toward higher PREX1 expression in HPV-positive tumors may also be biologically relevant. HPV-associated cervical carcinogenesis is characterized by activation of multiple oncogenic signaling pathways, including the PI3K/AKT pathway, which has been implicated in cervical tumor progression [22,23]. The tendency toward higher PREX1 expression in SILVA pattern C tumors may also be of interest. Since SILVA pattern C represents the most infiltrative growth pattern within the SILVA classification system and is associated with less favorable clinicopathological features, the observed trend is consistent with the recognized involvement of PREX1 in pathways related to cellular migration and invasion [24].

Interestingly, the observed trend toward higher PREX1 expression in SILVA pattern C tumors appears to contrast with our primary finding of increased PREX1 expression in surviving patients. One possible explanation is that PREX1 may influence specific aspects of tumor morphology or invasion without necessarily translating into an adverse clinical outcome. Alternatively, the biological effects of PREX1 may be context-dependent and influenced by additional molecular factors, including HPV-associated signaling pathways and the broader tumor microenvironment. Given the limited sample size and the absence of statistically significant differences according to the SILVA pattern, this apparent discrepancy should be interpreted with caution. Nevertheless, it highlights the complex and potentially multifaceted role of PREX1 in cervical adenocarcinoma biology.

A notable finding of our study is the association between higher PREX1 protein expression and favorable overall survival. While PREX1 typically functions as a pro-oncogenic Rac-GEF promoting migration and invasion in breast, prostate, and oral carcinomas, its clinical impact appears context-dependent. In estrogen receptor-positive breast cancers and certain gynecologic contexts, elevated PREX1 expression has been paradoxically linked to well-differentiated phenotypes or prolonged disease-free intervals under specific therapeutic regimens.

In endocervical adenocarcinoma, PREX1 upregulation may reflect intact intracellular trafficking or microenvironmental immune crosstalk rather than aggressive invasion. Furthermore, post-translational modifications, proteasomal degradation rates, or differential activation of downstream Rac/Rho signaling axes could decouple total protein abundance from functional oncogenic activity. Experimental functional assays and protein-level validation are essential to delineate whether PREX1 plays an organ-specific tumor-suppressive or homeostatic role in cervical glandular epithelium.

In addition to protein-level differences, survival-associated epigenetic alterations were identified in our cohort. Eight genes demonstrated significantly different methylation profiles between living and deceased patients. This pattern may indicate a predominance of epigenetic silencing events in tumors associated with adverse clinical outcomes. To further explore the potential biological relevance of these methylation changes, enrichment analysis was performed using the differentially methylated genes.

Interestingly, enrichment analysis identified a KLF9-associated regulatory motif as the only significantly enriched result. KLF9 belongs to the Krüppel-like factor family of zinc-finger transcription factors and functions as a regulator of cellular proliferation, differentiation, migration, apoptosis, and hormone-responsive transcriptional programs [25,26,27,28].

To directly address potential transcriptional regulation, we evaluated KLF9 transcript levels within our cohort and confirmed that KLF9 mRNA expression alone was not significantly associated with overall survival (*p* = 0.427; Figure 5B). This finding reinforces that the enriched KLF9 motif represents an in silico-predicted promoter binding signature derived from differentially methylated regions, rather than an active transcriptional driver in this cohort. Consequently, KLF9 should be viewed strictly as a novel candidate regulatory factor requiring future functional validation, such as ChIP-seq and luciferase reporter assays.

Previous studies have suggested that KLF9 may play an important role in gynecologic tumor biology. In endometrial cancer, KLF9 expression has been reported to be reduced compared with normal endometrium, and decreased KLF9 expression has been associated with enhanced proliferative and invasive capacity of tumor cells [26]. Moreover, KLF9 has been shown to interact with progesterone receptor-associated signaling pathways and to regulate downstream transcriptional networks involved in endometrial homeostasis and tumor progression [25,26]. Additional evidence suggests that KLF9 may regulate the expression of genes involved in tumor suppression and therapeutic response, including FBXO31, further supporting its role as a transcriptional regulator in gynecologic malignancies [29].

In cervical cancer, reduced KLF9 expression has been reported in peripheral blood samples from affected patients and was associated with tumor stage, metastatic status, and disease prognosis [30]. Collectively, these findings suggest that KLF9 may participate in multiple regulatory pathways relevant to tumor progression and clinical outcome.

This study provides an integrated preliminary assessment of survival-associated molecular features in endocervical adenocarcinoma; however, several important limitations must be acknowledged. First, the small total sample size (*n* = 27) and the marked numerical imbalance between survival groups (22 living vs. 5 deceased) significantly constrain statistical power, precluding multivariable Cox modeling to adjust for clinical parameters such as FIGO stage and age. Second, subgroup analyses regarding HPV-independent adenocarcinomas and SILVA Pattern A were restricted due to low case counts (*n* = 2 each). Third, functional wet-lab validation—such as in vitro assays or tissue microarray validation via immunohistochemistry (IHC)—was not performed. Fourth, as detailed above, external bioinformatic validation was precluded by the complete absence of independent, open-access proteomic datasets for cervical adenocarcinoma. Finally, the observed KLF9 transcription factor motif enrichment represents an in-silico association without direct verification of KLF9 expression or activity. Consequently, these findings should be viewed strictly as exploratory and hypothesis-generating.

Third, subgroup analyses regarding HPV-independent adenocarcinomas and SILVA Pattern A were restricted due to low case counts (*n* = 2 each), preventing robust molecular stratification between HPV-associated and HPV-independent pathways. Fourth, functional wet-lab validation—such as in vitro knockdown/overexpression assays or in situ tissue microarray validation via immunohistochemistry (e.g., PREX1 IHC)—was not performed. Finally, the observed KLF9 transcription factor motif enrichment represents an in-silico association without direct verification of KLF9 expression or activity. Consequently, these findings should be viewed strictly as exploratory and hypothesis-generating, warranting future validation in larger, multi-center tissue cohorts.

## Figures and Tables

**Table 1:** Survival-associated differentially methylated genes.

Gene	Higher Methylation Group	Genomic Location/Region	Promoter CpG Island Status	*p*-Value	*q*-Value	mRNA Correlation (r)
**MCEMP1**	Deceased	Chr 19q13.2	Promoter/CpG Island	1.53 × 10^−6^	0.0119	−0.34 (*p* = 0.08)
**WDR89**	Living	Chr 14q11.2	Promoter/Shore	1.71 × 10^−6^	0.0119	−0.28 (*p* = 0.15)
**RNASE2**	Deceased	Chr 14q11.2	Promoter/CpG Island	2.23 × 10^−6^	0.0119	−0.41 (*p* = 0.03)
**RINL**	Deceased	Chr 19q13.2	Body/Non-island	3.63 × 10^−6^	0.0145	−0.19 (*p* = 0.33)
**CCL17**	Deceased	Chr 16q13	Promoter/CpG Island	5.09 × 10^−6^	0.0163	−0.38 (*p* = 0.05)
**THBD**	Deceased	Chr 20p11.2	Promoter/CpG Island	6.53 × 10^−6^	0.0168	−0.45 (*p* = 0.02)
**SFXN5**	Deceased	Chr 2p13.1	Promoter/CpG Island	7.37 × 10^−6^	0.0168	−0.31 (*p* = 0.11)
**PRR19**	Deceased	Chr 19p13.2	Promoter/Shelf	1.46 × 10^−5^	0.0291	−0.22 (*p* = 0.27)

## Data Availability

The data analyzed in this study are publicly available through The Cancer Genome Atlas (TCGA) program and can be accessed via the cBioPortal for Cancer Genomics platform (https://www.cbioportal.org/ accessed on 31 July 2026).
